# Gastric metastasis subsequent to renal cell carcinoma surgery: a case report

**DOI:** 10.3389/fonc.2025.1624528

**Published:** 2025-08-18

**Authors:** Junqiao Yao, Xiangyu Meng, Yanmei Zhu, Dong Yang, Jun Zhang, Chao Wang, Yuanlin Liu, Tao Zhang

**Affiliations:** Gastric Cancer Department, Liaoning Province Cancer Hospital & Institute (Cancer Hospital of China Medical University), Shenyang, Liaoning, China

**Keywords:** gastric metastasis, renal cell carcinoma, gastric cancer, immunohistochemical, comprehensive treatment

## Abstract

Gastric metastasis of renal cancer involves the dissemination of renal cancer cells to the stomach via hematogenous or lymphatic routes, leading to the formation of metastatic tumors. Renal cell carcinoma (RCC), a highly aggressive malignant tumor, exhibits a significant propensity for metastasis. Common metastatic sites include the lung, bone, liver, and brain. This report presents a case of gastric metastasis originating from renal cell carcinoma in a 62-year-old male patient who was diagnosed with clear cell renal cell carcinoma in the left kidney in March 2024. The gastric space-occupying lesion was confirmed through imaging studies, gastroscopy, and histopathological examination. This case is documented herein to enhance clinical awareness and improve diagnostic accuracy for this rare manifestation of RCC.

## 1 Introduction

**Figure 1:**
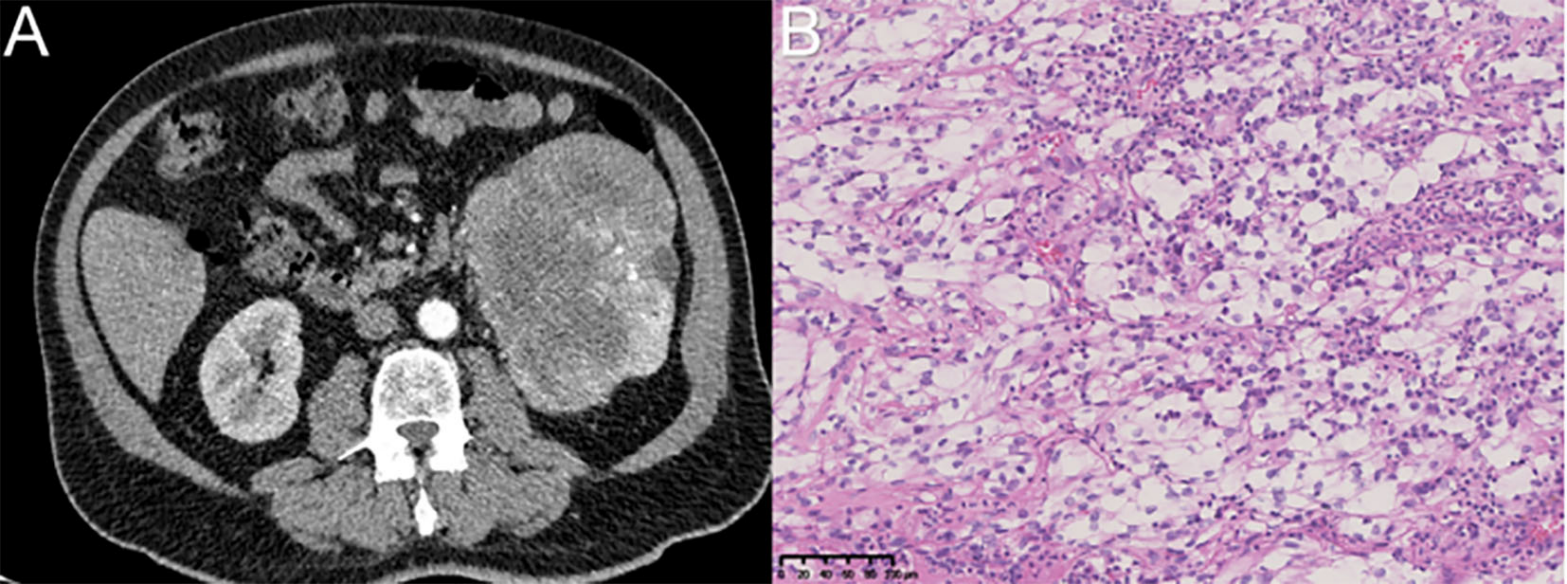
The diagnosis of renal cell carcinoma was confirmed through imaging via CT and postoperative immunohistochemical analysis. **(A)** Renal carcinoma was suspected based on imaging findings of the left kidney. **(B)** Immunohistochemical analysis (magnification, ×200) confirmed the diagnosis of clear cell renal cell carcinoma in the left kidney.

Gastric cancer (GC) is the fifth most common malignant tumor in the world (1). Due to its insignificant early symptoms and low early screening rate, most patients are in advanced stage when diagnosed. Gastric metastasis refers to the spread of malignant tumors from other sites to the stomach through hematogenous, lymphatic, or direct invasion. Cases of secondary gastric metastasis have been reported in patients with breast cancer, lung cancer, esophageal cancer, and other malignancies (2–4)., but in general, gastric metastasis is relatively rare and has a poor prognosis. The median survival time is usually 6–12 months, which mainly depends on the type of primary cancer and the sensitivity of systemic treatment. Here we present a patient with gastric metastasis 11 months after surgery for clear cell renal cell carcinoma, and discuss the imaging and pathological features of gastric metastasis of renal cell carcinoma (**Figure 1**).

## 2 Case report

A 62-year-old man was admitted to the hospital due to epigastric discomfort lasting for three days. Three days prior to admission, he developed epigastric discomfort accompanied by abdominal distention without any apparent precipitating factors. A gastroscopy performed at another hospital revealed a mass in the gastric fundus, and subsequent pathological analysis indicated mucosal low-adhesion carcinoma. Subsequently, the patient sought further treatment at our institution. Eleven months earlier, he had been diagnosed with left renal cell carcinoma at our center and underwent left nephrectomy. The postoperative pathological examination revealed a tumor measuring 14 × 10 × 10 cm, with a confirmed diagnosis of clear cell renal cell carcinoma grade II. No definitive vascular tumor embolus, perineural invasion, or involvement of the adrenal gland or perihilar renal tissue was observed. Upon presentation at our center, CT imaging suggested possible pulmonary metastasis; however, the patient declined further diagnostic confirmation via needle biopsy for personal reasons. According to the medical consultation, pazopanib was initially administered orally at a daily dose of 4 tablets (800 mg) from April 2024 to December 2024. Subsequently, the dosage was adjusted to 3 tablets (600 mg) per day due to observed fluctuations in blood pressure and creatinine levels.

In addition, the patient had a documented history of hypertension for over two years, which was effectively managed with oral antihypertensive medications. Upon admission to our center, his blood pressure was recorded as 130/80 mmHg. Laboratory tests conducted at our facility revealed the following: carbohydrate antigen 19-9 (CA19-9): 13.84 U/mL (within normal range, 0–34.0 U/mL), carbohydrate antigen 125 (CA125): 29.6 U/mL (within normal range, 0–35.0 U/mL), carcinoembryonic antigen (CEA): 2.41 ng/mL (within normal range, 0–5.0 ng/mL), neuron-specific enolase (NSE): 11.41 µg/L (within normal range, 0–16.3 µg/L), and alpha-fetoprotein (AFP): 3.07 ng/mL (within normal range, 0–7.0 ng/mL).

**Figure 2:**
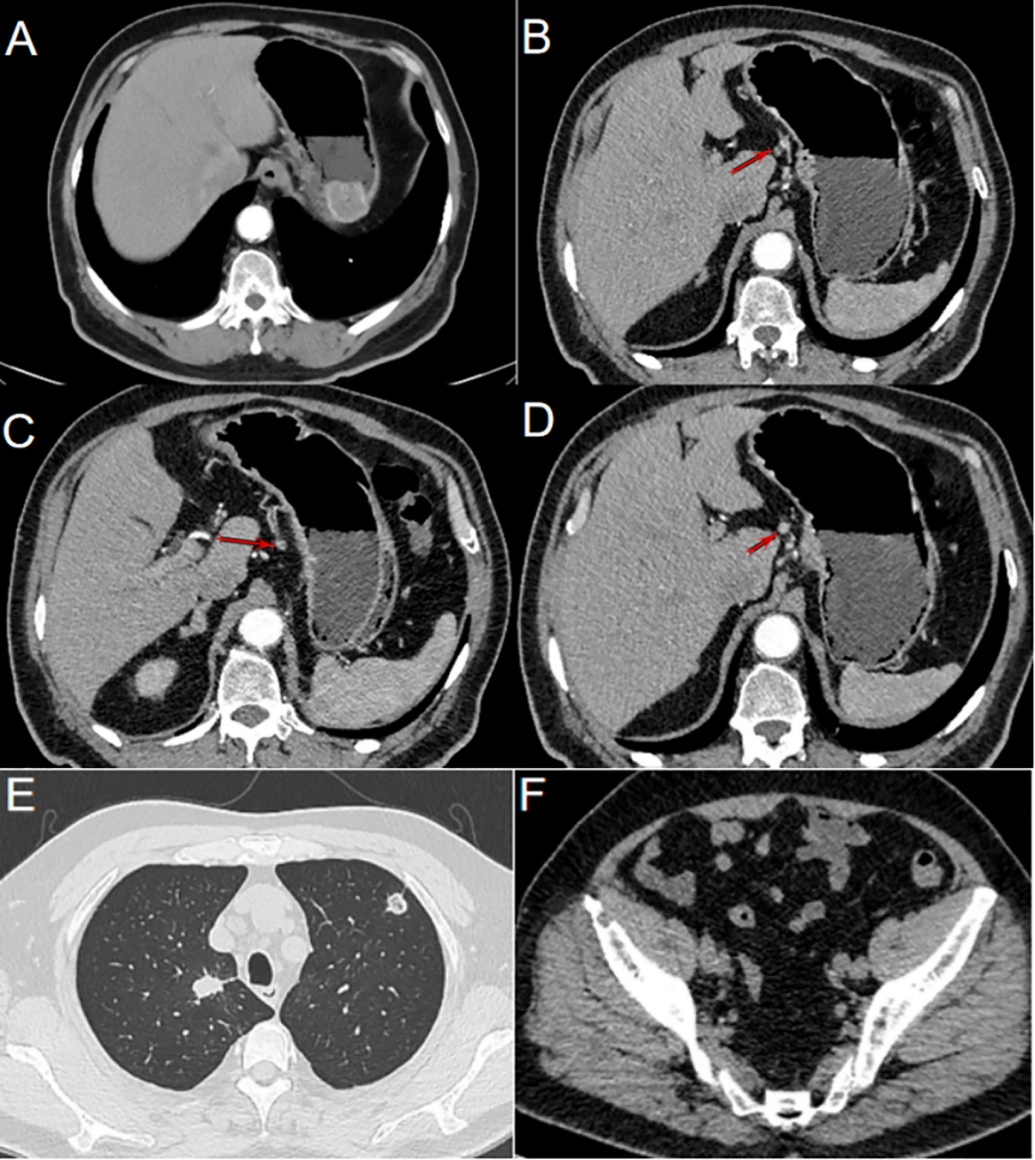
Gastric metastasis following renal cell carcinoma resection. **(A)** CT imaging revealed a prominent mass located in the fundus of the stomach. **(B)** Lymph nodes clustered along the lesser curvature of the stomach (as indicated by the arrow). **(C)** Enlarged lymph nodes were observed in the lesser curvature of the stomach (as indicated by the arrow). **(D)** Round, enlarged lymph nodes in the lesser curvature of the stomach cannot be ruled out (as indicated by the arrow). **(E)** Solid nodules of varying sizes were observed in both lungs, and metastatic tumors were suspected. **(F)** Bone destruction observed in the right iliac crest, with the possibility of metastasis not excluded.

The patient underwent thin-slice computed tomography (CT) of the lungs, three-dimensional reconstruction of enhanced CT of the stomach, and enhanced CT of the pelvis. These imaging studies identified a well-defined mass in the fundus of the stomach, measuring approximately 32 mm × 32 mm × 17 mm, with local protrusion of the gastric contour and several enlarged lymph nodes surrounding the stomach. Additionally, multiple nodules of varying sizes were observed in both lungs, showing enlargement compared to prior imaging studies performed at our center. Bone destruction in the right iliac crest was noted, raising suspicion for possible metastasis (**Figure 2**).

**Figure 3:**
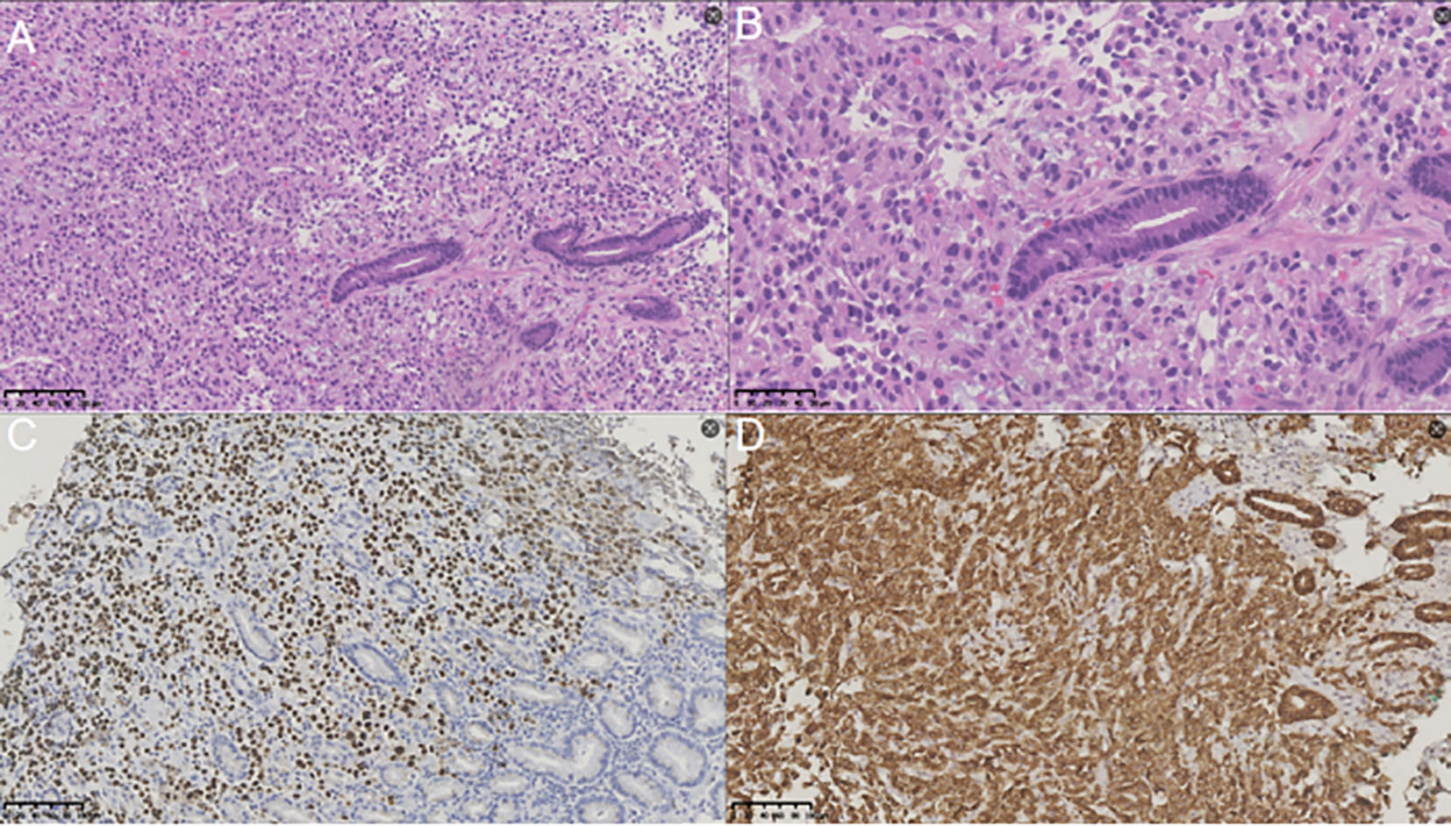
Results of immunohistochemical staining analysis. **(A)** Hematoxylin and eosin (H&E) staining (magnification, ×200) revealed that the gastric lesion was a metastasis of renal carcinoma. **(B)** Hematoxylin and eosin (H&E) staining (magnification, ×400) revealed that the gastric lesion was a metastasis of renal carcinoma. **(C)** Immunohistochemical staining at a magnification of 200× demonstrated PAX-8 positivity in the gastric mass. **(D)** Immunohistochemical staining at a magnification of 200× demonstrated CA-IX positivity in the gastric mass.

The patient’s gastroscopic pathology from other hospitals was consistent with poorly differentiated carcinoma. In conjunction with immunohistochemical findings and medical history obtained in our hospital, the possibility of metastatic renal cell carcinoma could not be excluded. To determine the origin of the lesion, a gastroscopic biopsy was conducted at our center. The pathological report indicated the presence of cancerous tissue in the gastric fundus, suggestive of metastatic renal cell carcinoma. Immunohistochemical staining demonstrated positivity for Vim, CA-IX, PAX-8, Ki-67, and CD10. Based on these histological characteristics, we concluded that the gastric fundus carcinoma represented metastasis from renal cell carcinoma (**Figure 3**).

Considering the patient’s overall clinical condition, surgical intervention is currently not recommended. Instead, the patient is undergoing comprehensive treatment with axitinib, denosumab, and toripalimab. Per consultation recommendations, axitinib is administered orally twice daily at 5 mg per dose, and toripalimab is administered intravenously at 240 mg per cycle. Denosumab is delivered via subcutaneous injection once every 21 days, starting one week after the initiation of immunotherapy. To date, the patient has completed one cycle of this regimen without reporting significant adverse effects.

Blood tests will be conducted prior to each treatment cycle to monitor changes in tumor markers and hematological parameters, exclude potential contraindications to treatment, and assess the patient’s current physiological status. Furthermore, contrast-enhanced CT scans of the thorax and whole abdomen will be performed every two treatment cycles to evaluate alterations in lesions and lymph nodes and to determine the efficacy of the ongoing treatment. Following 3 to 4 treatment cycles, a gastroscopy will be performed to obtain a biopsy and evaluate the status of the lesion. Adjustments to the examination protocol and treatment plan will be made based on the patient’s individual treatment response, with the objective of developing an optimal treatment strategy tailored to the specific clinical scenario.

## 3 Discussion

In general, among the various subtypes of renal cell carcinoma (RCC), clear cell RCC constitutes the largest proportion and is the most prevalent subtype (5), accounting for approximately 80% of all RCC cases. The remaining subtypes are collectively categorized as non-clear cell RCC. For patients with non-metastatic RCC, the primary treatment options include radical or partial nephrectomy (6). In cases where RCC is locally advanced, adjuvant therapy with sunitinib or pazopanib may be employed to mitigate the risk of recurrence. The mainstay treatment strategy for metastatic RCC involves a multimodal approach centered on targeted therapy and immunotherapy. Approximately 30% of RCC patients develop metastases, and the 5-year survival rate for those with metastatic disease is only 14% (7). Among RCC patients who have undergone surgical intervention, about 20-30% experience postoperative recurrence and metastasis (8).

In recent years, the advancement of targeted therapy and immunotherapy has offered novel approaches for the comprehensive management of renal cell carcinoma (RCC). Nevertheless, treatment strategies for RCC patients with varying histological subtypes and stages remain to be refined. The KEYNOTE-564 trial represents the first positive immune checkpoint inhibitor (ICI) adjuvant study in RCC. This trial evaluated patients with intermediate-to-high risk (pT2N0M0 grade 4 or sarcomatoid, or pT3N0M0 any grade) or high-risk (pT4N0M0 any grade, or pT3N1M0 any grade) clear-cell RCC (ccRCC) who had undergone radical or partial nephrectomy. These patients were treated with pembrolizumab for 12 months (9). Study findings demonstrated a significant improvement in disease-free survival (DFS) among patients receiving adjuvant pembrolizumab compared to placebo. However, 20.7% of patients in the pembrolizumab group discontinued treatment due to adverse events, while 7.4% required high-dose glucocorticoid therapy for immune-related adverse effects (10).

The primary metastatic routes of renal cell carcinoma (RCC) encompass hematogenous metastasis, lymphatic metastasis, and direct invasion. The most frequent metastatic sites for RCC are the lung, bone, liver, adrenal gland, and brain (11), whereas gastric metastasis is relatively uncommon. Gastric metastasis is not only rare in RCC but also across various other cancer types. A literature review revealed that gastric metastasis has been reported in breast cancer, lung cancer, esophageal cancer, and other malignancies. There are differences in the diagnosis and treatment approaches for primary gastric cancer and gastric metastatic cancer. The diagnosis of primary gastric cancer is primarily based on endoscopic biopsy and imaging evaluation, with treatment typically centered on surgical resection and chemotherapy. In contrast, the diagnosis of gastric metastatic cancer requires identifying the primary tumor site through immunohistochemical analysis, and its management should be guided by the systemic treatment strategy tailored for the original cancer type. In cases where pathological findings are inconclusive, a multidisciplinary team (MDT) consultation is recommended to enhance diagnostic accuracy and optimize therapeutic planning.

When gastric metastasis occurs, it is typically associated with epigastric discomfort or upper gastrointestinal bleeding. For early-stage gastric cancer, surgical resection is generally considered the preferred treatment option (12). However, if a patient has a history of advanced cancer as mentioned above, further pathological biopsy and immunohistochemical analysis must be conducted to confirm whether the stomach represents the primary lesion. Resection of metastatic lesions typically does not confer a survival benefit (13). If gastric lesions are identified as metastatic, systemic therapy should be prioritized to enhance patient survival. Systemic therapy is directed at systemic diseases and is capable of controlling multifocal metastases. It helps avoid surgical trauma and reduces the risk of complications. As a form of precision medicine, it aims to prolong survival time and improve overall quality of life.

A case of gastric metastasis from renal cell carcinoma (RCC) was reported at a certain medical center. The patient was initially diagnosed with RCC and underwent radical right nephrectomy. Fourteen years later, due to upper gastrointestinal bleeding, a gastroscopy was performed, revealing polyps in the gastric fundus. Histological examination confirmed metastasis of RCC, and additional metastatic lesions were identified in the ribs and lungs (14). In cases of upper gastrointestinal bleeding similar to this one, aside from implementing necessary hemostatic measures, it is crucial to conduct tissue biopsies for comprehensive pathological evaluation and to perform imaging studies to further confirm the diagnosis.

When gastric metastasis of RCC occurs, it is frequently associated with metastasis in other organs. Approximately 80% of RCC patients exhibit lung and bone metastases at the time of diagnosis with gastric metastasis, while isolated gastric metastasis is exceedingly rare (15). Prior studies have summarized and reported a total of 42 cases of RCC-related gastric metastasis (16), comprising 11 cases of isolated gastric metastasis and 31 cases involving multiple organ metastases including the stomach. Patients with RCC and gastric metastasis are at a significantly elevated risk of recurrence and further metastasis; therefore, meticulous consideration must be given to the formulation of treatment strategies. A comprehensive approach should be adopted to devise a more effective systemic treatment plan rather than proceeding hastily with surgical resection.

## 4 Conclusion

In conclusion, pathology serves as a pivotal component in the diagnosis and classification of renal cell carcinoma (RCC), while imaging modalities such as computed tomography (CT) and magnetic resonance imaging (MRI) also play essential roles in diagnosing RCC. In clinical practice, when a patient with confirmed RCC presents with a gastric tumor, the possibility of gastric metastasis from RCC should be considered. Further diagnostic evaluation, including relevant imaging studies and gastroscopic biopsy for pathological immunohistochemical analysis, should be conducted to confirm the diagnosis.

## Data Availability

The original contributions presented in the study are included in the article/Supplementary Material. Further inquiries can be directed to the corresponding author.
